# Identification of an autophagy-related gene signature that can improve prognosis of hepatocellular carcinoma patients

**DOI:** 10.1186/s12885-020-07277-3

**Published:** 2020-08-17

**Authors:** Xingxing Huo, Jian Qi, Kaiquan Huang, Su Bu, Wei Yao, Ying Chen, Jinfu Nie

**Affiliations:** 1 University of Science and Technology of China, Hefei, China; 2 Hefei Cancer Hospital, Chinese Academy of Sciences, Hefei, China; 3 Anhui Province Key Laboratory of Medical Physics and Technology, Institute of Health and Medical Technology, Hefei Institutes of Physical Science, Chinese Academy of Sciences, Hefei, China; 4 Experimental Center of Clinical Research, the First Affiliated Hospital of Anhui University of Chinese Medicine, Hefei, China

**Keywords:** Autophagy, HCC, Autophagy-related genes, Molecular signature, Drug sensitivity

## Abstract

**Background:**

Autophagy is a programmed cell degradation mechanism that has been associated with several physiological and pathophysiological processes, including malignancy. Improper induction of autophagy has been proposed to play a pivotal role in the progression of hepatocellular carcinoma (HCC).

**Methods:**

Univariate Cox regression analysis of overall survival (OS) was performed to identify risk-associated autophagy-related genes (ARGs) in HCC data set from The Cancer Genome Atlas (TCGA). Multivariate cox regression was then performed to develop a risk prediction model for the prognosis of 370 HCC patients. The multi-target receiver operating characteristic (ROC) curve was used to determine the model’s accuracy. Besides, the relationship between drug sensitivity and ARGs expression was also examined.

**Results:**

A total of 62 differentially expressed ARGs were identified in HCC patients. Univariate and multivariate regression identified five risk-associated ARGs (HDAC1, RHEB, ATIC, SPNS1 and SQSTM1) that were correlated with OS in HCC patients. Of importance, the risk-associated ARGs were independent risk factors in the multivariate risk model including clinical parameters such as malignant stage (HR = 1.433, 95% CI = 1.293–1.589, *P* < 0.001). In addition, the area under curve for the prognostic risk model was 0.747, which indicates the high accuracy of the model in prediction of HCC outcomes. Interestingly, the risk-associated ARGs were also correlated with drug sensitivity in HCC cell lines.

**Conclusion:**

We developed a novel prognostic risk model by integrating the molecular signature and clinical parameters of HCC, which can effectively predict the outcomes of HCC patients.

## Background

Hepatocellular carcinoma (HCC), which accounts for 75–85% of liver cancer cases, is considered the sixth most common malignancy and the fourth with cancer-related death worldwide [1]. The main causes of liver cancer are chronic infection with hepatitis B/C virus, exposure to aflatoxin, alcohol abuse, and obesity [2]. HCC is usually associated with poor outcomes because the treatment of HCC could be effective only when diagnosed at early stages [3]. The prognosis of HCC is currently dependent on histopathological parameters and the tumor staging system. However, such traditional approaches might not be adequate for the accurate prediction of clinical outcomes in HCC patients. Therefore, it is imperative to identify more robust and accurate prognostic indicators that can help clinicians optimize therapeutic strategies.

Autophagy is a natural regulatory mechanism by which cells remove nonessential and dysfunctional components. It is a dynamic process that includes the induction of autophagosomes, their nucleation, double membrane growth and closure, and finally, fusion with the lysosome, which leads to disintegration of the engulfed materials [4]. Abnormal autophagy has been associated with the pathogenesis of a variety of diseases, including malignant tumors [5]. In tumors, autophagy can exert opposite environment-dependent effects, which can lead to either suppression or promotion of tumor growth [6]. Indeed, while autophagy is considered an essential gatekeeper for restricting early tumorigenesis in multiple tissues [7], defective autophagy has been shown to promote tumor proliferation in several tissues [8]. In fact, deficiency in autophagy could lead to the release of arginase I from the liver, which leads to the degradation of circulating arginine. Hence, autophagy might maintain cancer growth through circulating arginine [9].

Recent studies have reported that autophagy plays a crucial role in the pathogenesis of HCC. Indeed, autophagy levels are noticeably higher in HCC tumor tissues, compared with adjacent normal tissues. In addition, the invasion of peripheral areas by HCC tumors has been associated with higher levels of autophagy in HCC cancer cells [10]. Autophagy promotes HCC cell proliferation through the induction intracellular ATP via mitochondrial oxidative phosphorylation [11]. Despite that several indexes have been proposed for HCC prognosis [12–14], little studies have considered autophagy-related genes (ARGs) for the prediction of clinical outcomes in HCC patients. Lin et al. reported that an expression signature for ARGs related to survival prediction for HCC patients [15]. Due to individual differences in HCC patients and the expression levels of relevant genes, additional predictors of HCC prognosis are needed that are not influenced by other clinical characteristics.

## Methods

### Patients information

RNA-seq data and clinical information of HCC patients were obtained from The Cancer Genome Atlas (TCGA) database and The International Cancer Genome Consortium (ICGC) dataset. Genes associated with autophagy were extracted from the Human Autophagy Database (HADb), an autophagy-dedicated database that provides information on human genes involved in autophagy.

### Functional annotation of differentially expressed ARGs

The R package *EdgeR* was used to perform differential gene expression analysis on ARGs in the TCGA data. ARGs exhibiting a log2 fold-change > 1 in HCC, compared with non-tumor tissues, and an adjusted *P* < 0.05 were considered to be significantly altered. Gene ontology (GO) and Kyoto Encyclopedia of Genes and Genomes (KEGG) enrichment analysis was performed using DAVID web-tool (The Database for Annotation, Visualization and Integrated Discovery) to unveil biological attributes and signaling pathways associated with the differentially expressed ARGs. The *GOplot* and *ClusterProfiler* R packages were used for visualization of the selected enriched ontologies and pathways.

### Construction of the prognostic risk model

Univariate cox regression analysis was used to identify differentially expressed ARGs associated with overall survival (OS) in HCC patients from the TCGA-LIHC data set. The identified OS-related ARGs were then included in a multivariate cox regression analysis to identify potential independent prognostic ARGs in HCC patients. The obtained prognostic ARGs were used to construct a risk score model. The regression coefficients in the linear formula were used as relative weights of ARG genes in the multivariate model. A risk score was calculated for each patient, a median value was identified for all patients, and HCC patients were then divided into low risk (score below the median) and high risk (score above the median) groups. The high and low risk groups were stratified and visualized using Kaplan-Meier (K-M) survival curves and analyzed for statistical significance using the log-rank test. The ARG-based risk score was finally included in a multivariate cox regression of OS to identify its prognostic value in HCC patients.

### Evaluation of the prognostic capacity of the model

The *survivalROC* package was used to analyze the prognostic value of the ARG-based risk model in R environment. The Receiver Operating Characteristic (ROC) curve was used to check the prognostic efficiency of the risk model in survival prediction. An area under the ROC curve (AUC) was used to measure the prognostic efficiency of the model.

### Statistical analysis

Data management and statistical analysis were performed using the R software. Plots were created using the R software and GraphPad Prism v7. K-M curves were plotted, and a log-rank test was applied to check for statistical differences between survival curves. A *P* < 0.05 was used as a threshold for statistical significance.

## Results

### Differentially expressed ARGs

**Fig. 1:**
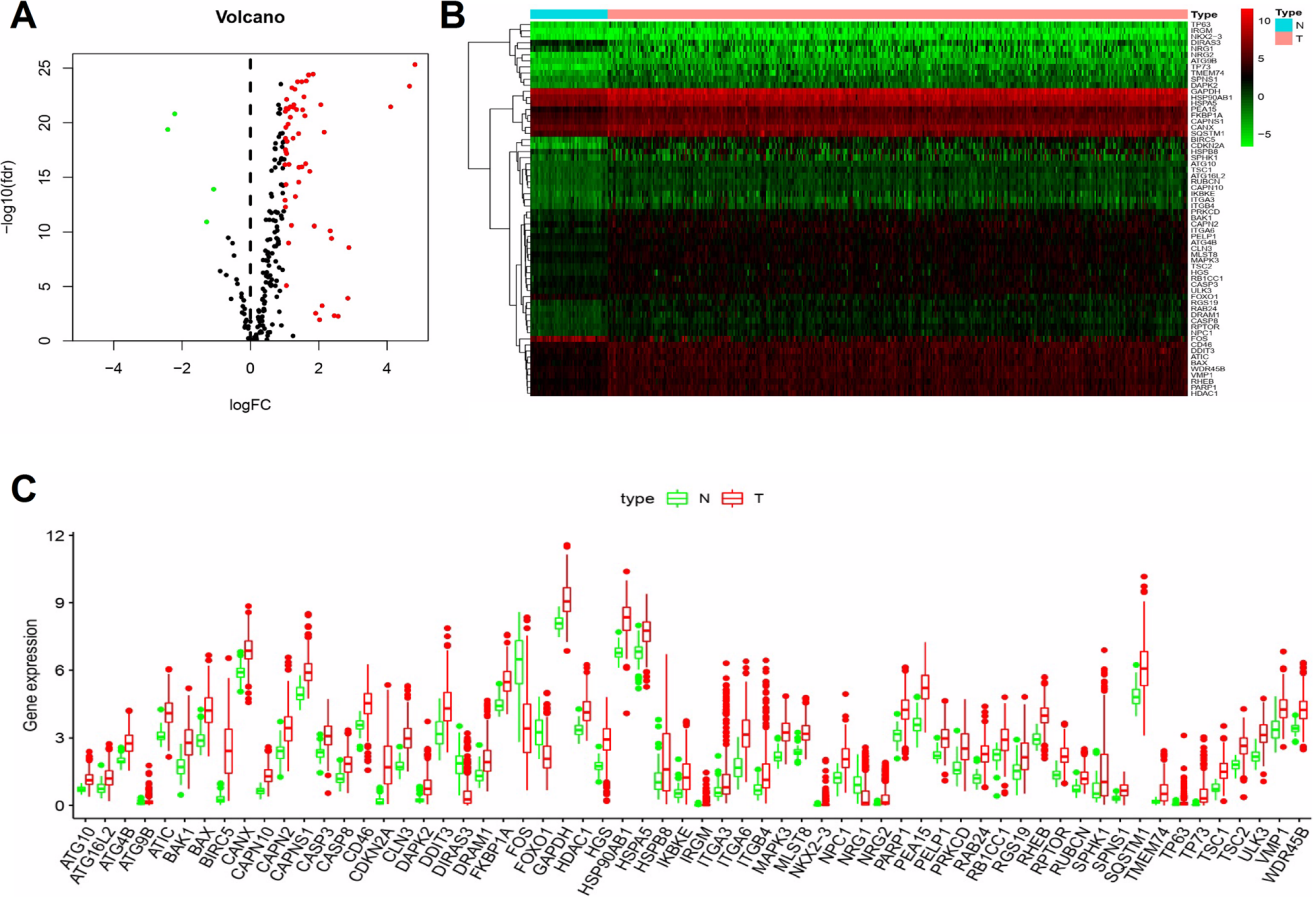
Differentially expressed autophagy-related genes (ARGs) between hepatocellular carcinoma (HCC) and normal samples. **a** A total of 222 HCC-related ARGs are represented in the volcano plot. Red points indicate upregulated ARGs, while green points represent downregulated ARGs in HCC, compared with normal tissue samples. **b** Hierarchical clustering of 62 differentially expressed ARGs in HCC, compared with normal tissue samples. Intensities of red and green colors indicate higher or lower gene expression, respectively. **c** The expression profile of ARGs in HCC and corresponding non-tumor samples. Red boxplots represent gene expression distribution in tumor tissue samples, while green boxplots represent gene expression distribution in normal tissue samples. These plots were created using R software v3.6.1

A total of 232 ARGs were identified using the HADb. A total of 370 patients with primary HCC had their clinical data and gene expression profiles available on the TCGA database (Table 1). Differential gene expression analysis identified 62 ARGs, including 58 upregulated and 4 down-regulated ARGs (Fig. 1a-b). Figure 1c shows the expression profiles of the differentially expressed ARGs in HCC and non-tumor tissue samples.

**Table 1:** Clinical data of 370 HCC patients

Clinical parameters	Variable	Total (370)	Percentages (%)
Age	< 65	221	59.73%
	≥65	149	40.27%
Gender	Female	121	32.70%
	Male	249	67.30%
Histological grade	G1	55	14.86%
	G2	177	47.84%
	G3	120	32.43%
	G4	13	3.51%
	unknow	5	1.35%
Pathological stage	Stage I	172	46.49%
	Stage II	84	22.70%
	Stage III	85	22.97%
	Stage IV	5	1.35%
	unknow	24	6.49%
TMN			
T staging	T1	182	49.19%
	T2	92	24.86%
	T3	80	21.62%
	T4	13	3.51%
	TX/unknow	3	0.81%
N staging	N0	252	68.11%
	N1	4	1.08%
	NX/unknow	114	30.81%
M staging	M0	266	71.89%
	M1	4	1.08%
	MX	100	27.03%
Survival status	Dead	125	33.78%
	Alive	245	67.40%

### Functional enrichment analysis

**Fig. 2:**
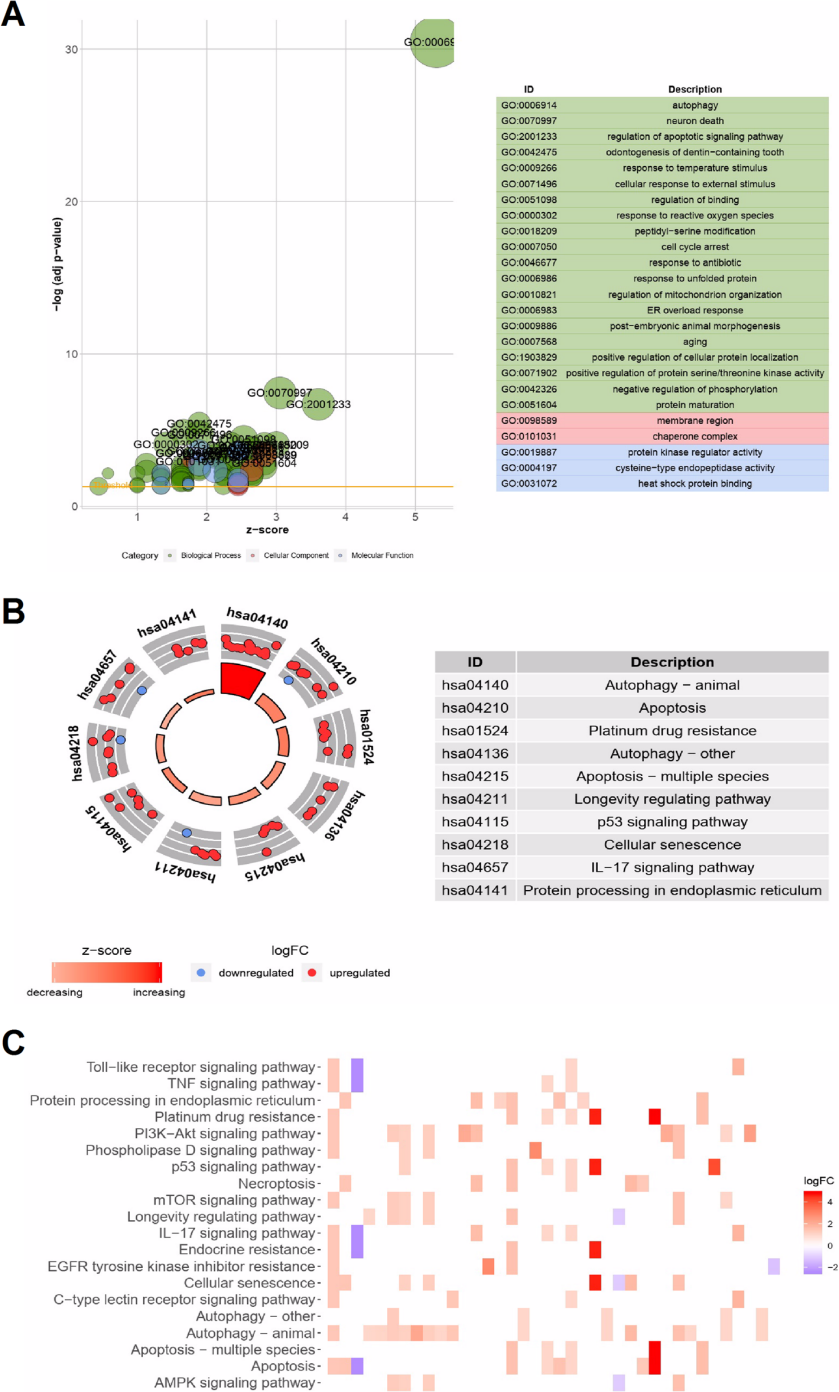
Functional enrichment analysis of the differentially expressed ARGs. **a** The enriched Gene ontology (GO) terms are shown in a bubble plot. The size of the displayed circles is proportional to the number of genes assigned to the term. Green circles represent biological process terms, red circles represent cellular component terms, and blue circles represent molecular function terms. **b** The outer circle shows a scatter diagram the logFC allocated to each term. **c** The heatmap shows the relationship between differentially expressed ARGs and the enriched Kyoto Encyclopedia of Genes and Genomes (KEGG) pathways. The heatmap colors represent logFC value of each gene in HCC, compared with normal samples. These plots were created using R software v3.6.1

Enrichment analysis was used to identify functional GO terms and KEGG pathways associated with the 62 differentially expressed ARGs in HCC samples. The GO biological processes associated with these genes were “process utilizing autophagic mechanism”, and “macroautophagy”, while the GO molecular functions associated with these genes were “protein kinase regulator activity”, “cysteine-type endopeptidase activity”, and “heat shock protein binding”. Regarding cellular components, the top two enriched GO terms were “region” and “chaperone complex” (Fig. 2a). On the other hand, enrichment analysis on showed that the differentially expressed ARGs were mainly associated with the following KEGG pathways: autophagy, apoptosis, platinum drug resistance, cellular senescence, p53 signaling pathway, IL-17 signaling pathway, and protein processing in endoplasmic reticulum. An enrichment z-score < 0 indicated that the relationship with the pathways could be reduced (Fig. 2b). The heatmaps in Fig. 2c show the relationship between the differentially expressed ARGs and the enriched pathways.

### Identification of risk-associated ARGs

**Fig. 3:**
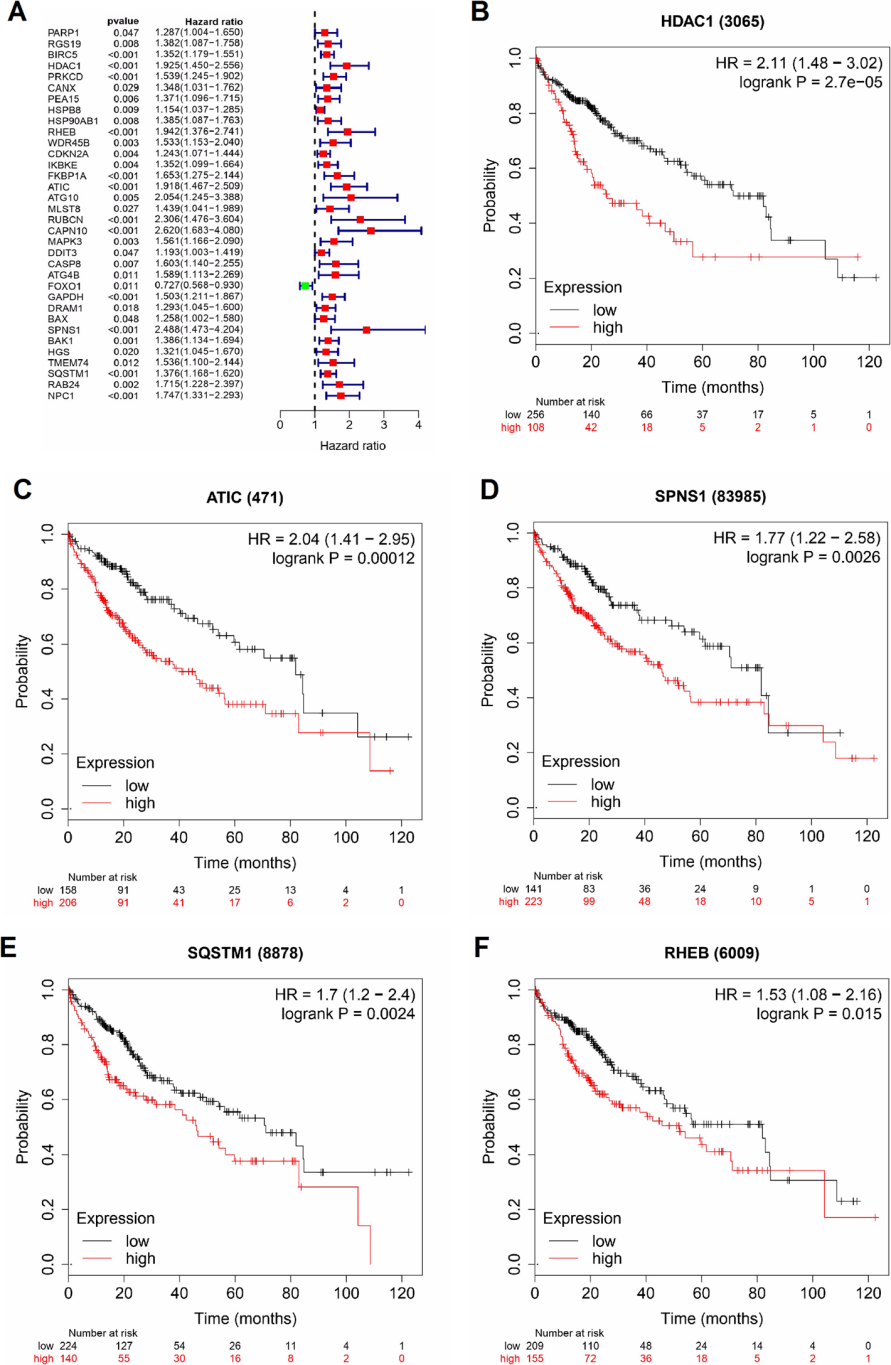
Univariate cox regression and Kaplan-Meier (K-M) survival curves of the differentially expressed ARGs. **a** A total of 34 ARGs were correlated with overall survival (OS) times of HCC patients (*P* < 0.05). **b-f** K-M curves showing the OS probability of patients stratified based on their expression of HDAC1, RHEB, ATIC, SPNS1 and SQSTM1, respectively

The correlation between expression levels of the 62 differentially expressed ARGs and OS was evaluated using the TCGA HCC data set. Univariate cox regression was first used to identify potential prognostic differentially expressed ARGs in the HCC patients. The analysis showed that 34 ARGs had their expression levels correlated with OS (Fig. 3a). Multivariate cox proportional hazard regression analysis was then performed order to construct a prognostic model that can efficiently predict outcomes of HCC patients. Interestingly, only 5 prognosis-related ARGs (HDAC1, RHEB, ATIC, SPNS1 and SQSTM1) were identified as potential independent risk factors (Table 2). The K-M analysis of OS showed that the high levels of HDAC1 and ATIC were strongly correlated with shorter OS time (HR = 2.11 and 2.04, respectively; 95% CI = [1.48–3.02] and [1.41–2.95], respectively; *P* < 0.001 for both; Fig. 3b, c). Similarly, high levels of SPNS1 and SQSTM1 were also associated with poor outcomes (HR = 1.77 and 1.70, respectively; 95% CI = [1.22–2.58] and [1.20–2.40], respectively; *P* < 0.01 for both; Fig. 3d, e). Likewise, high expression of RHEB was associated with shorter OS time of HCC patients (HR = 1.53, 95% CI = [1.08–2.16], *P* = 0.015; Fig. 3f). The two risk-associated ARGs (ATIC 1 and SPNS) associate with DFS in patients with HCC (*p* = 0.013 and *p* = 0.0018, respectively). The other 3 risk-associated ARGs (SQSTM1, RHEB and HDAC1) are not significantly associated with DFS (*p* = 0.087, 0.061 and 0.12 respectively) (Supplementary Fig. S1).

**Table 2:** Multivariate cox regression analysis data of the prognosis-related ARGs in HCC

Gene	coef	HR (95% CI)	***P***-value
HDAC1	0.42	1.52 (1.08–2.16)	0.017281
RHEB	0.54	1.72 (1.16–2.55)	0.006484
ATIC	0.62	1.85 (1.29–2.66)	< 0.001
SPNS1	1.37	3.92 (1.62–9.49)	0.002491
SQSTM1	0.21	1.23 (1.03–1.48)	0.025931

### Construction of a prognostic model using ARG genes

**Fig. 4:**
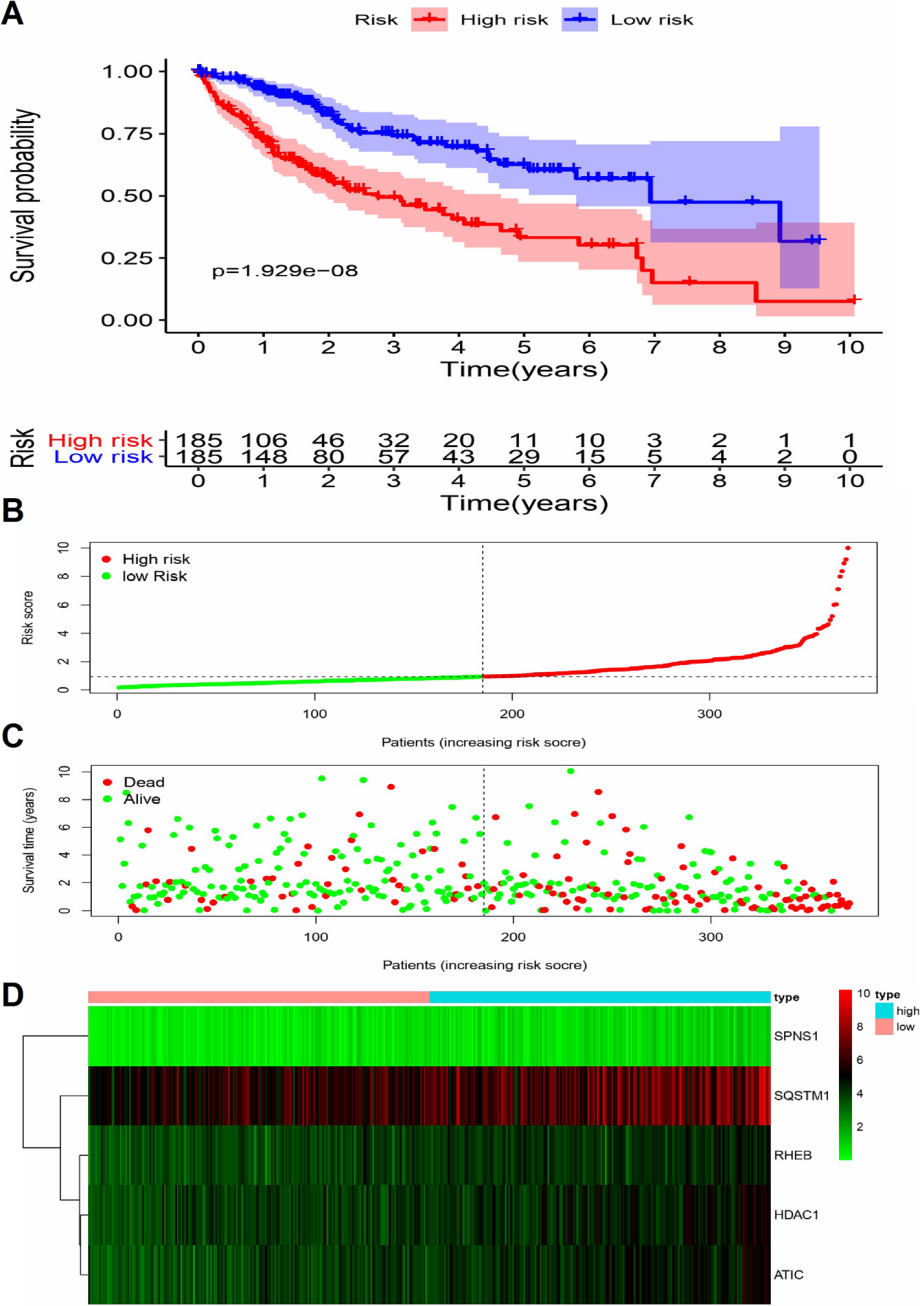
Correlation between the prognostic risk model and HCC patients’ survival probability. **a** K-M plot shows that patients in the high-score group had remarkably shorter OS time, compare with patients in the low-score group. **b-c** The distribution of risk score with patient’s survival outcomes. **d** Heatmap showing the expression profiles of the five risk-associated ARGs. These plots were created using R software v3.6.1

A linear regression model for the calculation of prognostic risk scores using expression levels (expr) of 5 ARGs weighted by their cox regression coefficients. The risk score was calculated using the following linear formula: riskScore = 0.4216 × exprHDAC1) + (0.5443 × exprRHEB) + (0.6171 × exprATIC) + (1.3652 × exprSPNS1) + (0.2082 × exprSQSTM1). A riskScore was calculated for each patient, and patients were then stratified for OS analysis into high and low risk groups relative to the median riskScore of all patients (*n* = 185). The K-M curve, along with the log rank test, indicated that the low-risk group exhibited favorable outcomes, while the high-risk group was associated with unfavorable outcomes (*p* < 0.001; Fig. 4a). The distribution and status of OS was then analyzed by ranking the risk scores (Fig. 4b-c). Figure 4d shows the expression profiles of risk-associated ARGs in high-risk and low-risk HCC patient groups.

### Significance of the ARG-based risk model as an independent risk factor

**Fig. 5:**
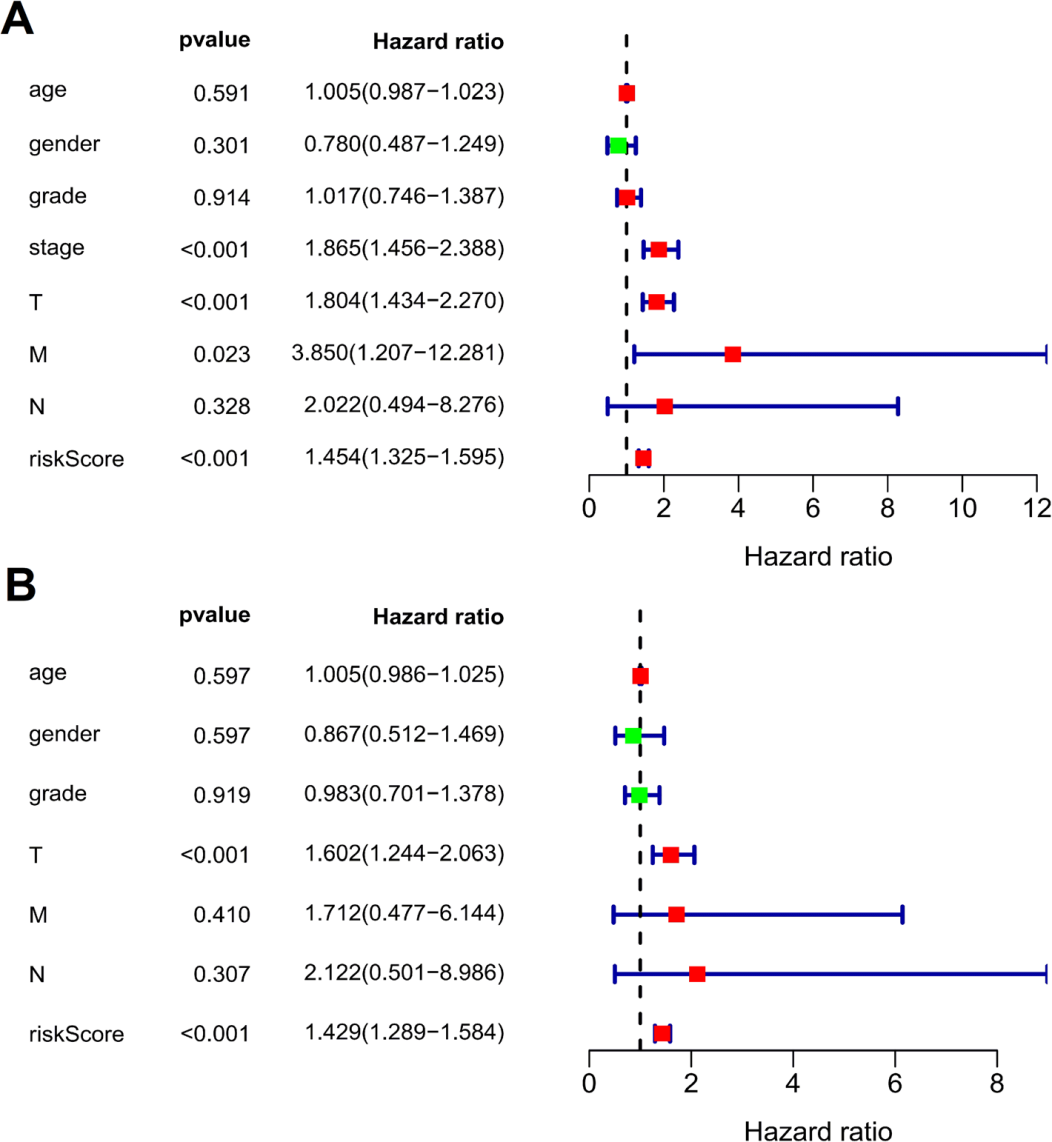
Univariate and multivariate regression analysis of overall survival (OS). (**a**) Univariate and (**b**) multivariate cox regression analyses show that the prognostic risk score was independently correlated with OS (*P* < 0.001). Forest plot showing the association between risk factors and OS in HCC patients. These plots were created using R software v3.6.1

The correlation of the clinical characteristics of patients and the riskScore with OS was then analyzed using univariate and multivariate regression analysis. Univariate cox regression analysis showed that the pathological stage, the T stage, the M stage and the riskScore were correlated with OS of HCC patients (*p* < 0.05; Fig. 5a). Of importance, multivariate cox regression analysis including clinical parameters and riskScores showed that only the riskScore was independently associated with OS of HCC patients (*p* < 0.001; Fig. 5b).

### The prognostic efficiency of the ARG-based risk model

**Fig. 6:**
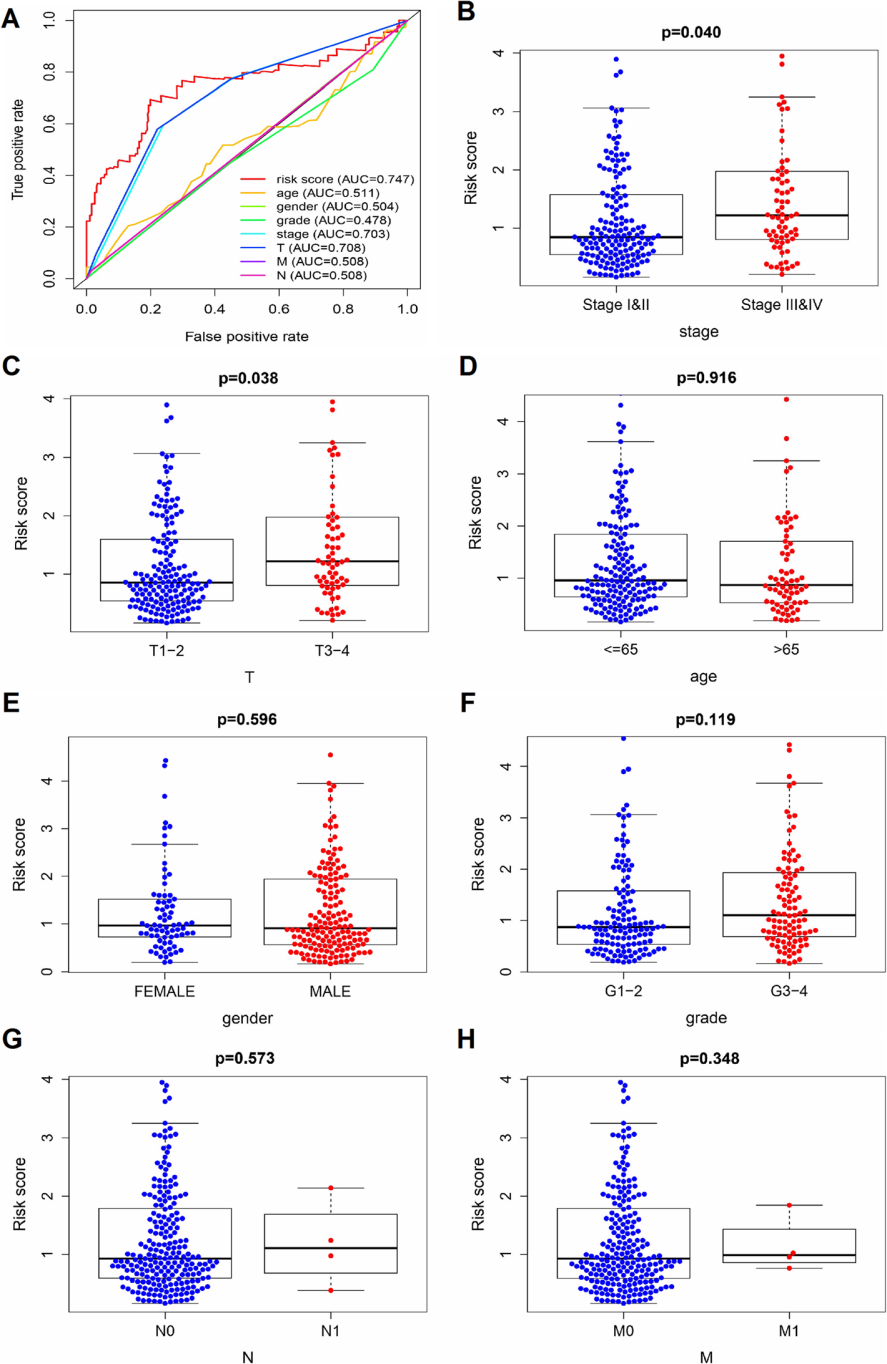
The prognostic efficiency of risk-associated ARGs in HCC patients. **a** Multi-target receiver operating characteristic (ROC) curve of the sensitivity and specificity of OS prediction based on the prognostic risk model in HCC patients. The clinicopathological significance of the risk score in HCC patients in relation with (**b**) cancer stages, (**c**) pathological T stages, (**d**) age, (**e**) gender, (**f**) histological grades, (**g**) pathological N stages, and (**h**) pathological M stages. These plots were created using R software v3.6.1

A multi-target ROC curve was performed to evaluate the prognostic efficiency of the risk model in the prediction of clinical outcomes in HCC patients. As shown in Fig. 6a, the AUC for the risk score was 0.747, which indicates a competitive performance.

The correlation between the risk score and clinical parameters was then analyzed. The results showed that the riskScore was higher in histological stages III-IV, compared with stages I-II (*P* = 0.040; Fig. 6b). In addition, the riskScore was higher in T3-T4 stages, compared with T1-T2 stages (*P* = 0.038; Fig. 6c). On the other hand, no differences in the riskScore were observed between patients > 65 and those ≤65 years old (*P* = 0.916), between male and female patients (*P* = 0.596), between grades G1-G2 and G3-G4 (*P* = 0.119), between stages N1 and N0 (*P* = 0.573), or between stages M1 and M0 (*P* = 0.348) (Fig. 6d-h).

### Verification of the ARG-based risk model in the testing group

**Fig. 7:**
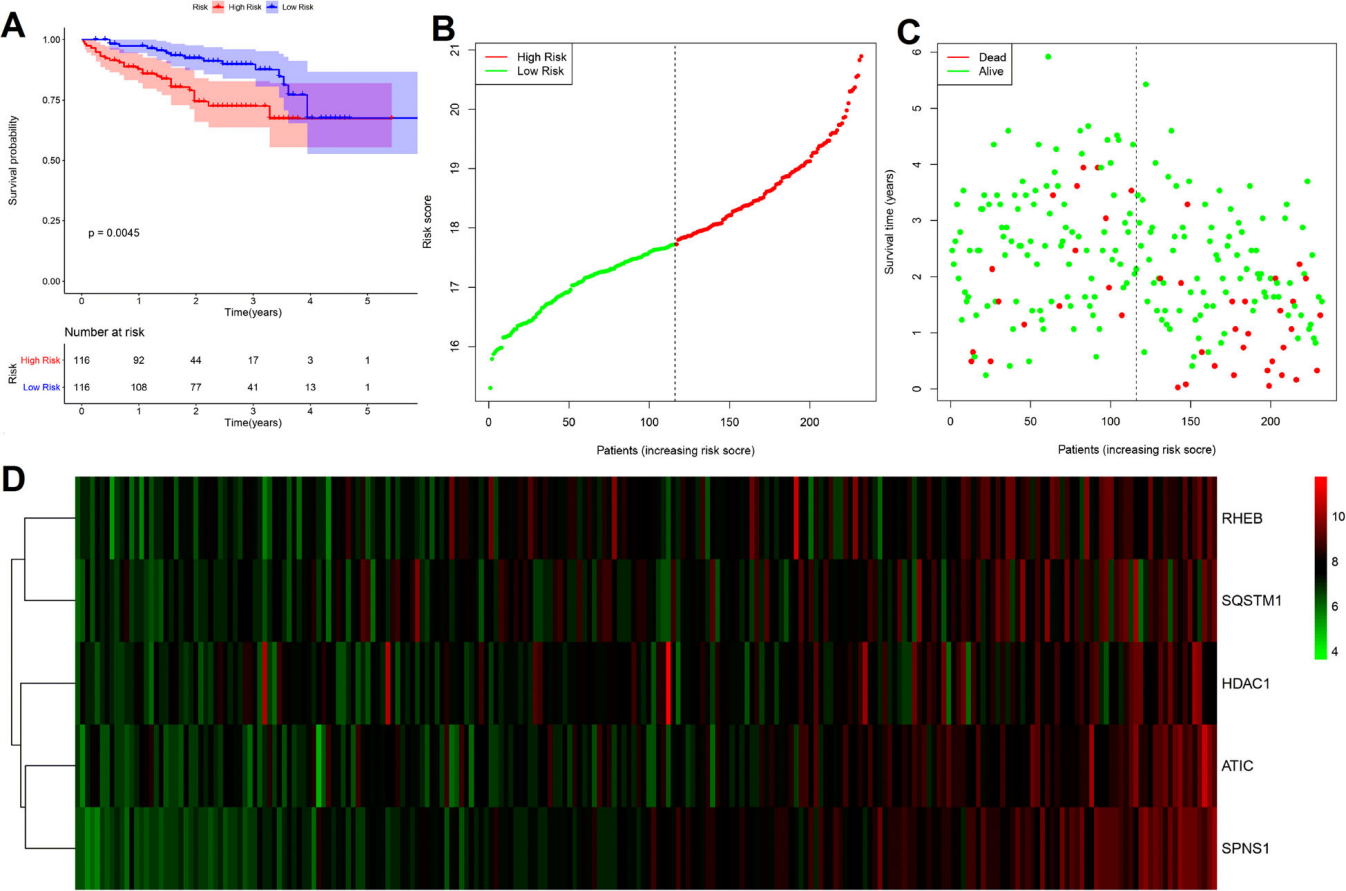
Verification of the prognostic risk model in the testing group. **a** K-M plot shows that patients in the high-risk group had inferior OS time, compare with patients in the low-risk group. **b-c** The distribution of risk score with patient’s survival outcomes. **d** Heatmap showing the expression profiles of the five risk-associated ARGs. These plots were created using R software v3.6.1

We further evaluated prognostic efficiency of the ARG-based risk model by analyzing the patients in the different liver cancer cohorts from ICGC dataset (https://dcc.icgc.org/releases/current/Projects/LIRI-JP). For 232 LIRI-JP samples, patients in high-risk group had inferior OS than patients in low-risk group (*p* = 0.0045) (Fig. 7a). The distribution and status of OS and expression profiles of risk-associated ARGs were also analyzed by ranking the risk scores in high-risk and low-risk HCC patient groups from ICGC dataset (Fig. 4b-d). Overall, the accuracy of ARGs-based risk model was confirmed in the independent validation liver cancer cohorts.

### The relationship of the drug sensitivity and risk ARGs

**Fig. 8:**
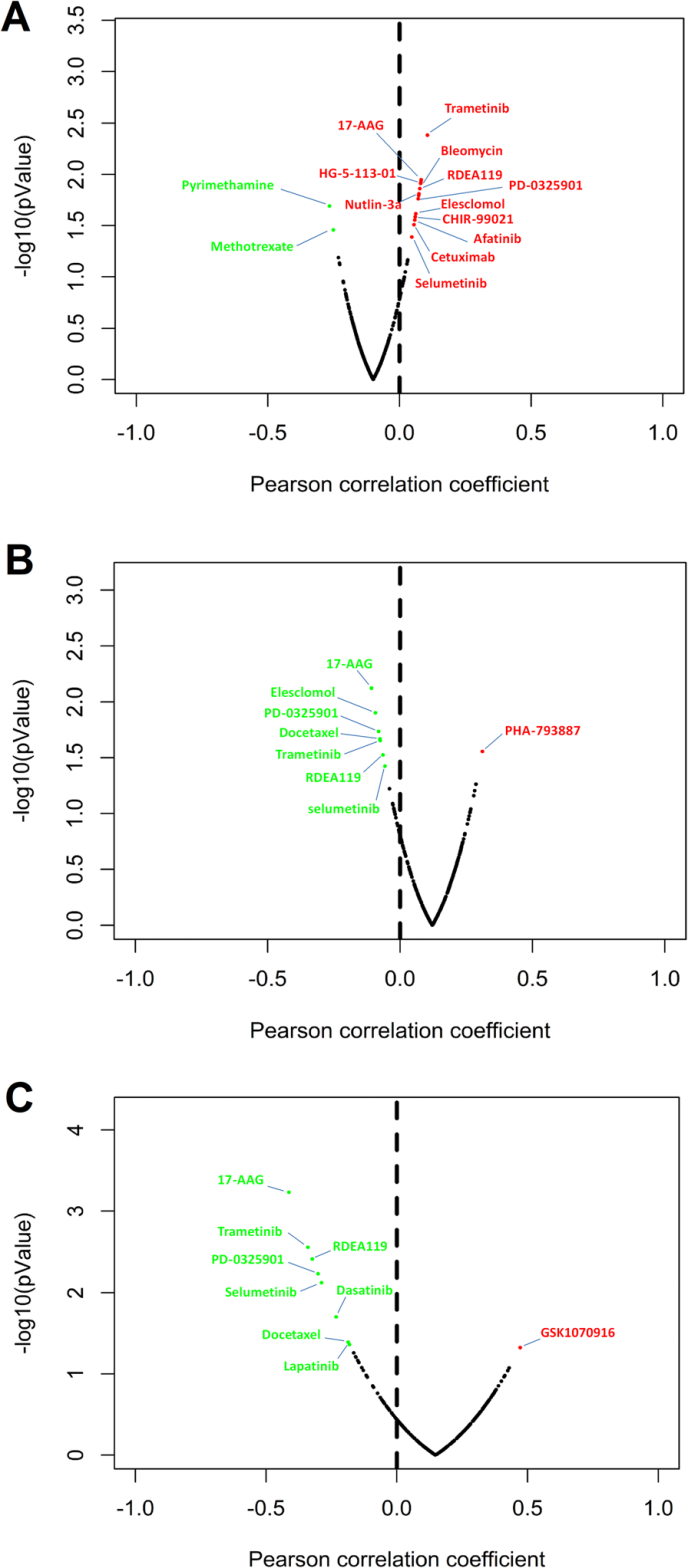
Relationship between risk-associated ARGs and drug resistance. The plots represent the correlation of (**a**) HDAC1, (**b**) RHEB and (**c**) SQSTM1 expression with the IC50 of several specific drugs in the liver cell lines. Red dots represent positive correlation between IC50 of the drug and the expression level of risk-associated ARGs, which indicates increased drug resistance with higher ARG expression (*p* < 0.05). Conversely, green dots represent negatively correlated drugs’ IC50 with the expression of risk-associated ARGs, indicating increased drug sensitivity with higher ARG expression (*p* < 0.05). These plots were created using R software v3.6.1

The relationship between drug sensitivity of 17 HCC cell lines and the relative expression levels of risk-associated ARGs was explored using data available from The Genomics of Drug Sensitivity of Cancer Database (GDSC). We further analyzed the correlation between the expression of HDAC1, RHEB and SQSTM1 with the IC50 of specific targeted drugs. We presumed that a positive correlation between the expression of these genes and the IC50 of the studied drugs would indicate a basis for developing drug resistance in HCC patients. In contrast, a negative correlation between risk-associated ARGs and IC50 would indicate higher drug sensitivity in HCC cell lines. High HDAC1 expression was associated with higher drug resistance (higher IC50) of HCC cell lines to Trametinib, 17-AAG, HG-5-113-01, Bleomycin, RDEA119, Nutlin-3a, PD-0325901, Elesclomol, CHIR-99021, Afatinib, Cetuximab and Selumetinib (*p* < 0.05), while it was associated with higher drug sensitivity (lower IC50) of HCC cell lines to Pyrimethamine and Methotrexate (*p* < 0.05; Fig. 8a).

On the other hand, higher RHEB expression resistance to ha-793,887 in HCC cell lines (*p* < 0.05), while it was associated with higher sensitivity to 17-AAG, Elesclomol, PD-0325901, Docetaxel, Trametinib, RDEA119 and Selumetinib (*p* < 0.05; Fig. 8b). Furthermore, higher SQSTM1 expression was associated with higher resistance of HCC cell lines to GSK1070916 (*p* < 0.05), while it was associated with higher sensitivity to other drugs such as 17-AAG, Trametinib, RDEA119, PD-0325901, Selumetinib, Dasatinib, Docetaxel and Lapatinib (*p* < 0.05; Fig. 8c).

## Discussion

The role of autophagy in maintaining genome integrity and cellular metabolism and homeostasis has been well demonstrated; however, its prognostic significance in human malignant tumors has not been fully explored [16, 17]. Autophagy can maintain the survival of tumor cells under stress, and hence, promote tumor progression. Despite that endogenous tumor factors and exogenous interventions to promote or suppress autophagy have been proposed as potential cancer treatments [4], autophagy-targeting cancer therapies remain controversial. Previous studies have reported that differential translation of autophagy-related transcripts may lead to malfunctional autophagosome in HCC cells [18]. Autophagy activation can promote the proliferation of HCC cells through JNK1/Bcl-2 signaling [19]. In addition, autophagy can promote metastasis through Wnt/β-catenin signaling [20] and via the induction of epithelial-mesenchymal transition [21]. Autophagy is considered an important mechanism of drug resistance by supporting the survival of cancer cells under metabolic and therapeutic stress [22]. In fact, sorafenib, the only drug approved for the treatment of advanced HCC, may promote autophagy in HCC cells through cellular protein networks. Luo et al. reported that the combination of PSMD10 and Atg7 could be used as a prognostic predictor in HCC patients receiving sorafenib therapy [23]. In addition, the expression level of the autophagy-related marker LC3 has been associated with poor outcomes in HCC patients receiving surgical resection [24].

In this study, the high-throughput transcriptomics data of HCC patients were analyzed to identify potential prognostic ARGs. A total of 62 ARGs were differentially expressed in HCC patient tumor samples, compared with normal tissues, including 58 up-regulated and 4 down-regulated genes. Univariate cox regression analysis was then performed on these genes to identify 34 ARGs that were correlated with OS of HCC patients. Of these, 5 risk-associated differentially expressed ARGs (HDAC1, RHEB, ATIC, SPNS1 and SQSTM1) were further identified using multivariate cox regression analysis and were used to construct a prognostic model for the risk-stratification of HCC patients based on a weighted risk score. Survival analysis showed that low-score groups exhibited better OS, compared with patients in high-score group. The multi-target ROC curve was then performed to validate the prognostic significance of the model, which was further analyzed for its correlation with clinical parameters of HCC patients. Previous work revealed that the 3 ARGs BIRC5, FOXO1 and SQSTM1 were associated with OS in HCC patients. HCC patients were stratified based on pathological stage [15]. Furthermore, results suggest that the risk score could significantly stratify HCC patients based on their histological and T-based staging systems.

HDAC1, a member of the histone deacetylase (HDACs) family, has been shown to play a crucial role in the epigenetic regulation of key oncogenes through the form a closed chromatin structure via histone deacetylation. A growing line of evidence has shown that HDAC1 could affect various oncogenic processes, such as cell proliferation and invasion, in multiple malignant tumors. The down-regulation of homeobox A10 has been shown to inhibit the proliferation of HCC cells and induce cell cycle arrest through the regulation of HDAC1 expression [25]. In addition, the transcription factor Yin-Yang 1 has been reported to reduce sensitivity of HCC cells to treatment by inducing HDAC1 expression [26]. Furthermore, miR-34a was demonstrated to inhibit cellular proliferation and induce apoptosis by down-regulation of HDAC1 expression in HCC cells [27]. A meta-analysis showed that high expression of HDAC1 is associated with poor OS in gastrointestinal and lung cancers, which indicates that HDAC1 may serve as a prognostic signature in these malignancies [28, 29].

Our results showed that RHEB, a key regulator of mTOR signaling, exhibited a high expression level in cancer samples, compared with normal and adjacent normal samples. Previous analysis of cancer cytogenetic and transcriptomic databases indicated that RHEB mRNA expression was up-regulated in different carcinoma histotypes and was associated with poor outcomes in multiple types of malignancies [30]. Besides, RHEB expression was associated with higher cancer stages, higher mortality, tumor differentiation and pathological satellites in patients with hepatitis B-related HCC [31, 32].

Previous studies have reported that ATIC is a bifunctional protease that catalyzes the last two steps in the purine biosynthesis pathway. Depletion of ATIC or suppression of its transformylase activity significantly decreased the survival rate of cells in clonogenic survival assays, which indicates that ATIC may promote the proliferation and migration in cancer cell lines [33]. Indeed, suppression of ATIC expression significantly inhibited the ability of HCC cells to proliferate and migrate through the regulation of the AMPK-mTOR-S6K1 signaling pathway. Therefore, in line with our results, the high expression of ATIC could be positively correlated with adverse prognosis in HCC patients [34].

SQSTM1 has been reported as a potential oncogene in various cancers, including HCC. p62, the gene product of SQSTM1, is a versatile protein that acts as an adaptor that induces the degradation of specific active molecules through autophagy [35]. Wei et al. reported that SQSTM1 contributes to the development of autophagy-deficient cancers via NF-kappaB pathway. Therefore, targeting autophagy and the autophagy-associated SQSTM1 gene expression could be exploited for developing more effective cancer treatments [36]. Indeed, phosphorylated SQSTM1/p62 has been shown to accumulate in the HCC tumor region, while its inhibitor may inhibit cell proliferation and resistance to anticancer agents [37]. Furthermore, multiple studies reported that SQSTM1 could serve as a novel prognostic biomarker in multiple cancers types, including nasopharyngeal carcinoma, lung cancer, oral squamous cell carcinoma, and HCC [38–41].

SPNS1 (Spinster homolog 1) is a hypothetical lysosomal H^+^-carbohydrate transporter that functions in late stage macroautophagy in vertebrates [42]. In this study, SPNS1 showed the greatest contribution to outcome predictions compared to the other 4 genes analyzed. In addition to OS, K-M analysis for DFS showed that high levels of SPNS1 also correlated with shorter DFS time (*p* = 0.013). Yanagisawa et al. reported that upregulation of SPNS1 regulates luminal solute compositions, thereby altering the subcellular distribution of lysosomes and the accumulation of p62 [43]. Dysregulation of autophagy lysosomes may promote the invasion and migration of HCC [44].

In the study presented here, we demonstrate the relationship between drug sensitivity of 17 HCC cell lines and the relative expression levels of risk-associated ARGs using the GDSC database. Even though many of these drugs are not in clinical use, identifying correlations between risk-associated ARGs and drug sensitivity may identify putative therapeutic biomarkers for further validation. Alterations in cancer genomes can influence clinical outcome to anticancer treatment. However, many cancer drugs already used and under development are not associated with specific genomic markers that can guide clinical application to maximize patient benefit [15]. In present study, we postulate that HDAC1 is a potential therapeutic target for HCC patients since high HDAC1 expression was associated with elevated drug resistance. By molecularly stratifying patient populations, drug sensitivity information can optimize the design of clinical trials and ultimate success of anticancer treatment.

## Conclusion

In conclusion, we have identified 5 prognostic risk-associated ARGs (HDAC1, RHEB, ATIC, SPNS1 and SQSTM1) by correlating the molecular signature of ARG with clinical outcomes of HCC patients. The identified risk-associated ARGs could provide a basis for the development of HCC therapeutic interventions via autophagy-related mechanisms. Of importance, we constructed a novel risk model that can robustly stratify HCC patients into risk groups. Nevertheless, further prospective experiments are required to further confirm the clinical value of this model in defining the optimal personalized targeted treatment.

## Supplementary information

**Additional file 1 Figure S1**. Disease free survival (DFS) curves of the differentially expressed ARGs in HCC patients. (A-E) K-M curves showing the DFS probability of patients stratified based on their expression of ATIC, SPNS1, SQSTM1, RHEB and HDAC1 respectively.

## Data Availability

The mRNA expression and clinical information of HCC were mined from TCGA-LIHC were available in the Genomic Data Commons Data Portal (National Cancer Institute, NIH, USA) repository. All relevant materials are provided in the manuscript.

## Abbreviations

HCC: Hepatocellular carcinoma
OS: Overall survival
DFS: Disease free survival
ARGs: Autophagy-related genes
TCGA: The Cancer Genome Atlas
ICGC: The International Cancer Genome Consortium
HADb: Human Autophagy Database
GO: Gene ontology
KEGG: Kyoto Encyclopedia of Genes and Genomes
K-M: Kaplan-Meier
ROC: Receiver Operating Characteristic
AUC: area under the ROC curve
expr: Expression levels
GDSC: Genomics of Drug Sensitivity of Cancer Database
